# Supplementary material for: Inhibitor-3 inhibits Protein Phosphatase 1 via a metal binding dynamic protein–protein interaction
Source: Nat Commun. 2023 Mar 31;14:1798. doi: 10.1038/s41467-023-37372-5 (PMC10066265; doi:10.1038/s41467-023-37372-5)
Supplement: Supplementary file 1 — Supplementary Information [file 41467_2023_37372_MOESM1_ESM.pdf]

Inhibitor-3 inhibits Protein Phosphatase 1 via a metal binding dynamic protein:protein interaction

Gautam Srivastava, Meng S. Choy, Nicolas Bolik-Coulon, Rebecca Page & Wolfgang Peti

Table of Contents

Supplementary Figure 1: $I3_{1\text{-}126}$ is an intrinsically disordered protein

Supplementary Figure 2: SPR sensorgrams for the reported PP1 and I3 constructs/variants

Supplementary Figure 3: I3 SILK motif interaction with PP1

Supplementary Figure 4: PP1 binding likely inhibits Caspase-3 mediated degradation of I3

Supplementary Figure 5: The effect of DTT and the role of $PP1_{C273S}$ in the inhibition of I3

Supplementary Figure 6: Molecular mechanisms of PP1 inhibition

Supplementary Table 1: SPR binding kinetics for the association of I3 variants with PP1α **S**

Supplementary Table 2: Data collection and refinement statistics

Supplementary Table 3: ITC thermodynamic parameters for I3-Zn^{2+} binding

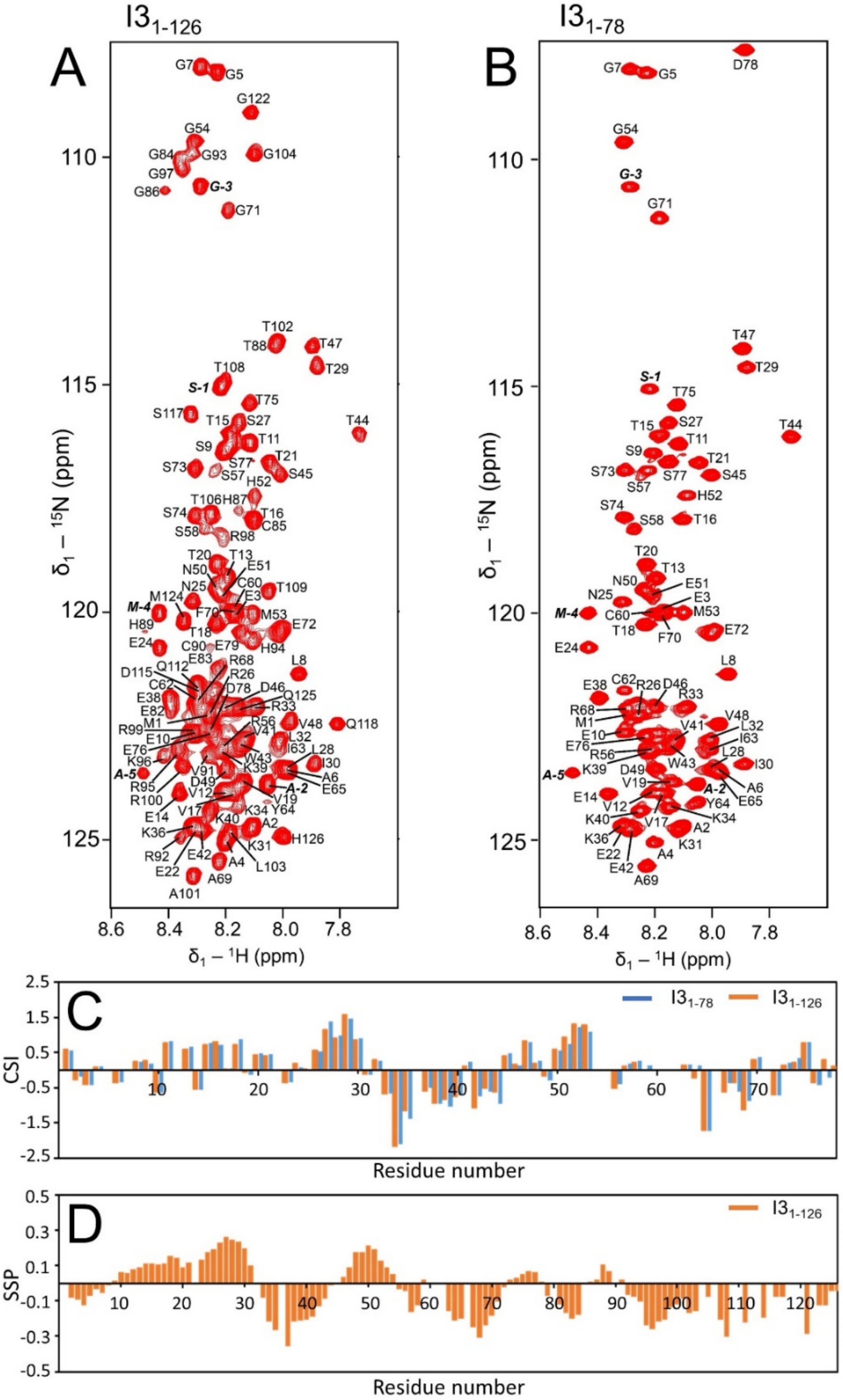

Supplementary Figure 1. $I3_{1\text{-}126}$ is an intrinsically disordered protein. A. Fully annotated 2D [^{1}H,^{15}N] HSQC spectrum of I3. **B.** Fully annotated 2D [^{1}H,^{15}N] HSQC spectrum of $I3_{1\text{-}78}$. **C**. Overlaid chemical shift Index (CSI) for I3 (orange) and $I3_{1\text{-}78}$ (blue). No CSI differences can be identified. **D**. secondary-structure propensity (SSP) data for I3 plotted vs. residue numbers. (SSP > 0, α helix; SSP < 0, β strand). Cα and Cβ chemical shifts were used to create the CSI and SSP plots in C/D (RefDB database).

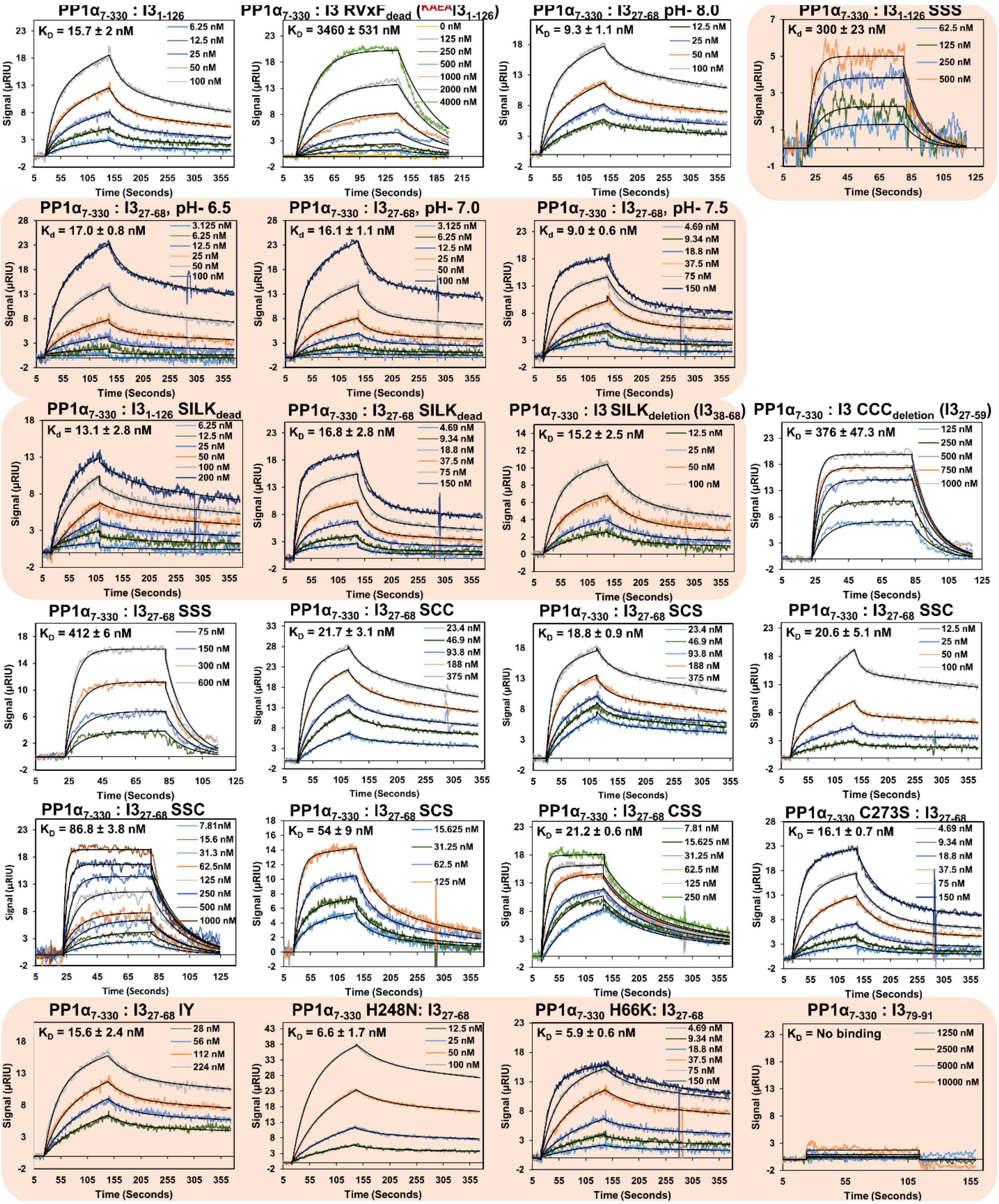

Supplementary Figure 2. SPR sensorgrams for the reported PP1 and I3 constructs/variants. Those with white backgrounds are also shown in the main manuscript; all sensorgrams are included here for ease of comparison.

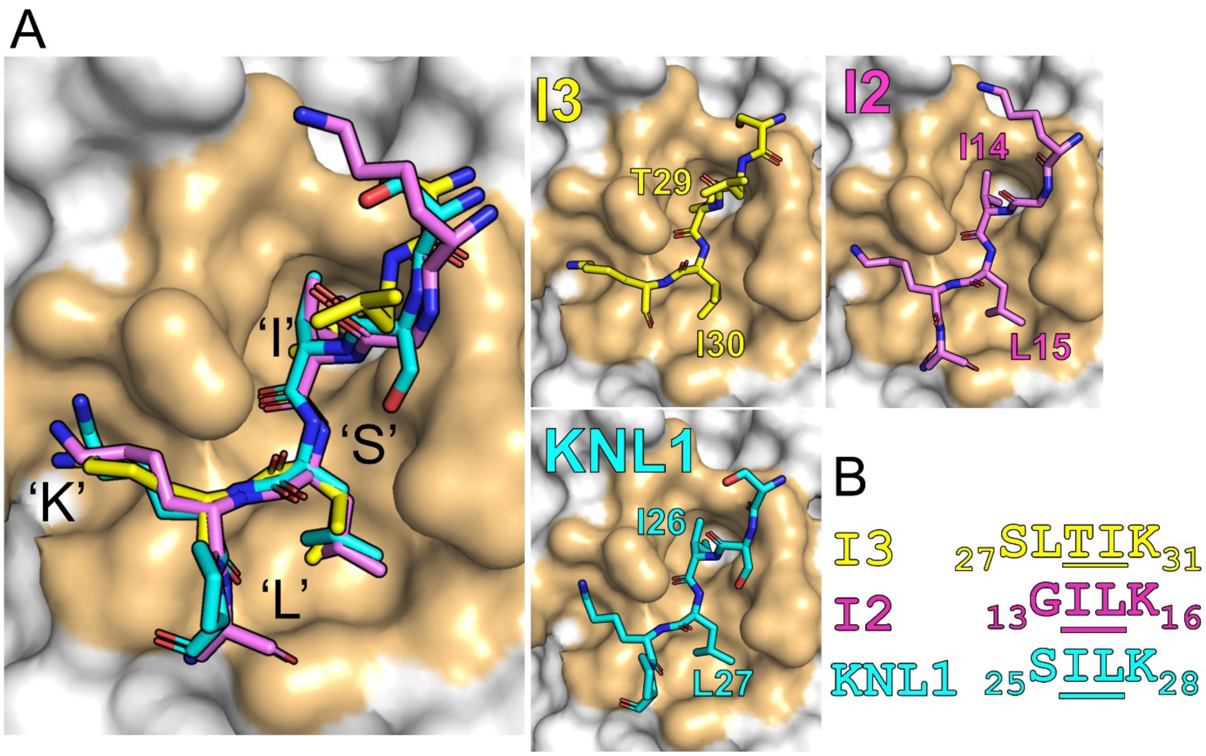

Supplementary Figure 3. I3 SILK motif interaction with PP1. A. The overlay of the SILK motifs of I3 (yellow), I2 (pink) and KNL1 (cyan); SILK PP1 binding pocket is shown as surface in light orange. Each interaction is also shown individually and labeled. **B**. Amino acid primary sequence of SILK motifs in I3, I2 and KNL1.

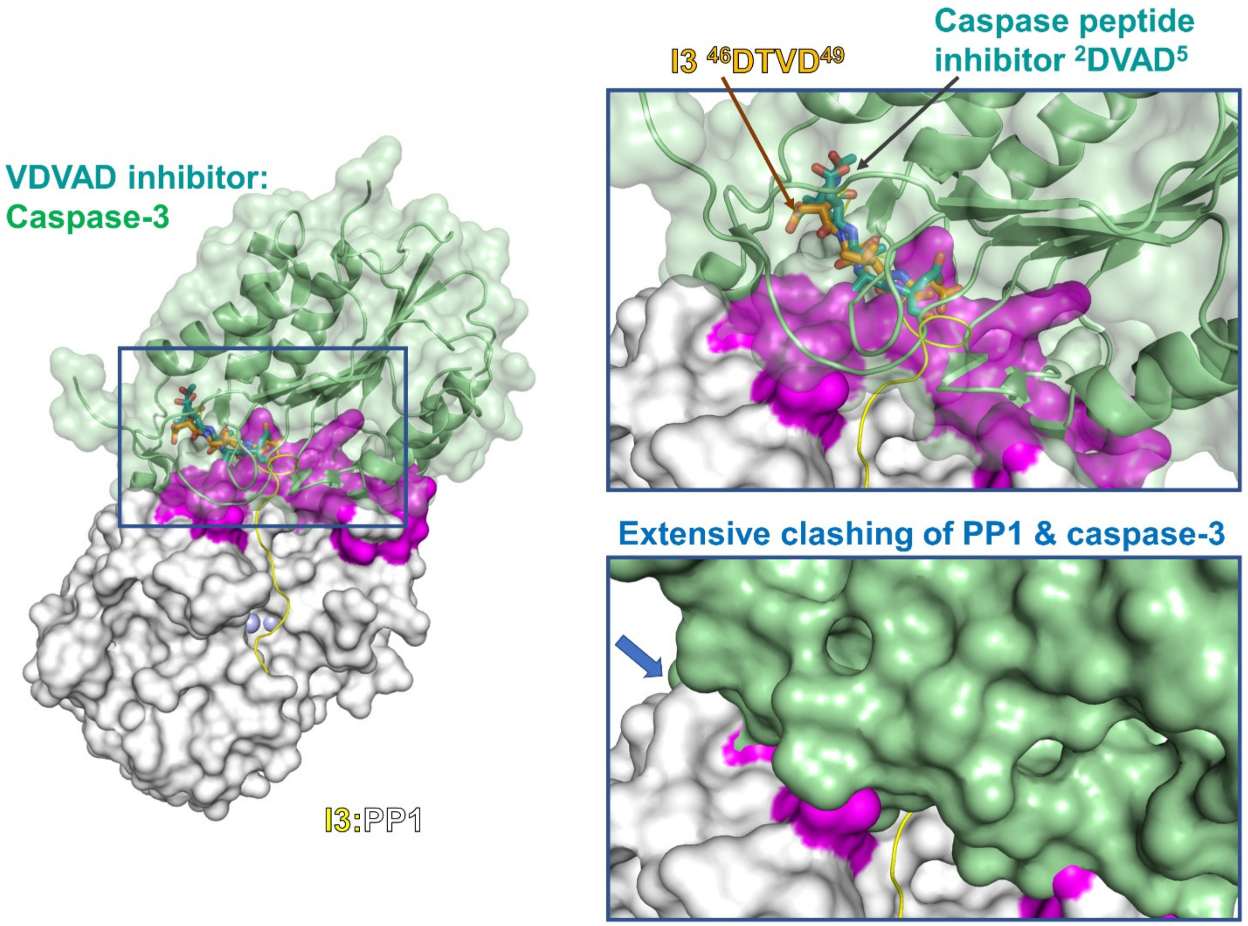

Supplementary Figure 4. PP1 binding likely inhibits Caspase-3 mediated degradation of I3. The complexes of I3:PP1 (I3 shown as a yellow ribbon with caspase cleavage sequence residues ^{46}DTVD^{49} shown as sticks and in orange; PP1 is shown as a grey surface; surface colored magenta reflects the region of caspase-3 that clashes with PP1 when the structures are superimposed) and VDVAD caspase inhibitor:Caspase-3 (VDVAD peptide shown as sticks and colored dark teal, caspase-3 shown as a transparent surface and cartoon in green; PDBID 2H65); crystal structures overlayed using the I3 ^{46}DTVD^{49} and Caspase peptide inhibitor ^{2}DVAD^{5} mainchain coordinates. Extensive clashing between PP1 and caspase-3 is observed, strongly suggesting that caspase-3 is likely unable to access the ^{46}DTVD^{49} cleavage sequence of I3 when I3 is bound to PP1.

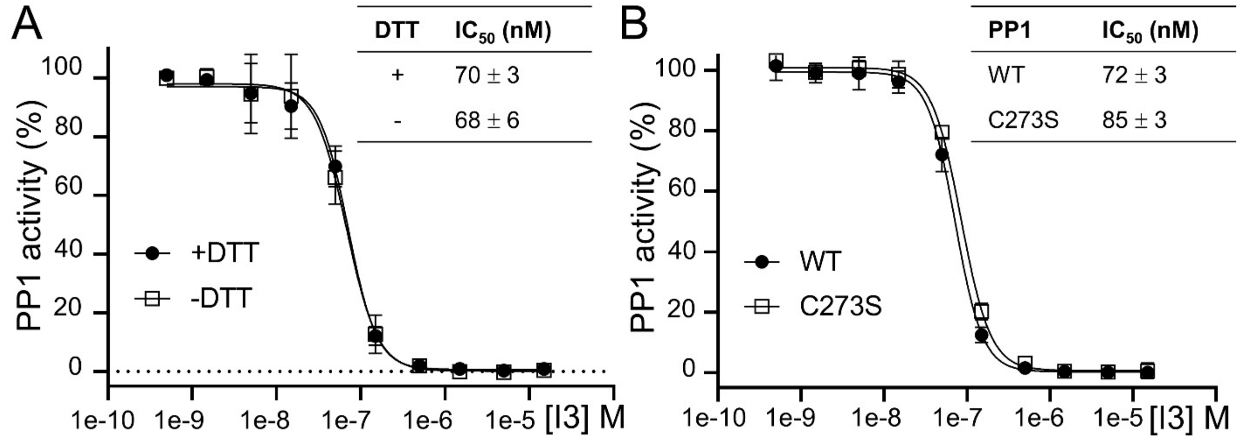

Supplementary Figure 5. The effect of DTT and the role of $PP1_{C273S}$ in the inhibition of I3. **A**. IC_{50} measurement of $I3_{27\text{-}68}$ in the presence or absence of 1.3 mM DTT. I3 was exchanged into buffer without 0.5 mM TCEP prior to the experiments. Data are presented as mean values +/- SD, n = 8 technical replicates. **B**. IC_{50} measurement of $I3_{27\text{-}68}$ with WT or $PP1_{C273S}$ (assays performed in buffer containing 1.3 mM DTT). Data are presented as mean values +/- SD, n = 8 technical replicates.

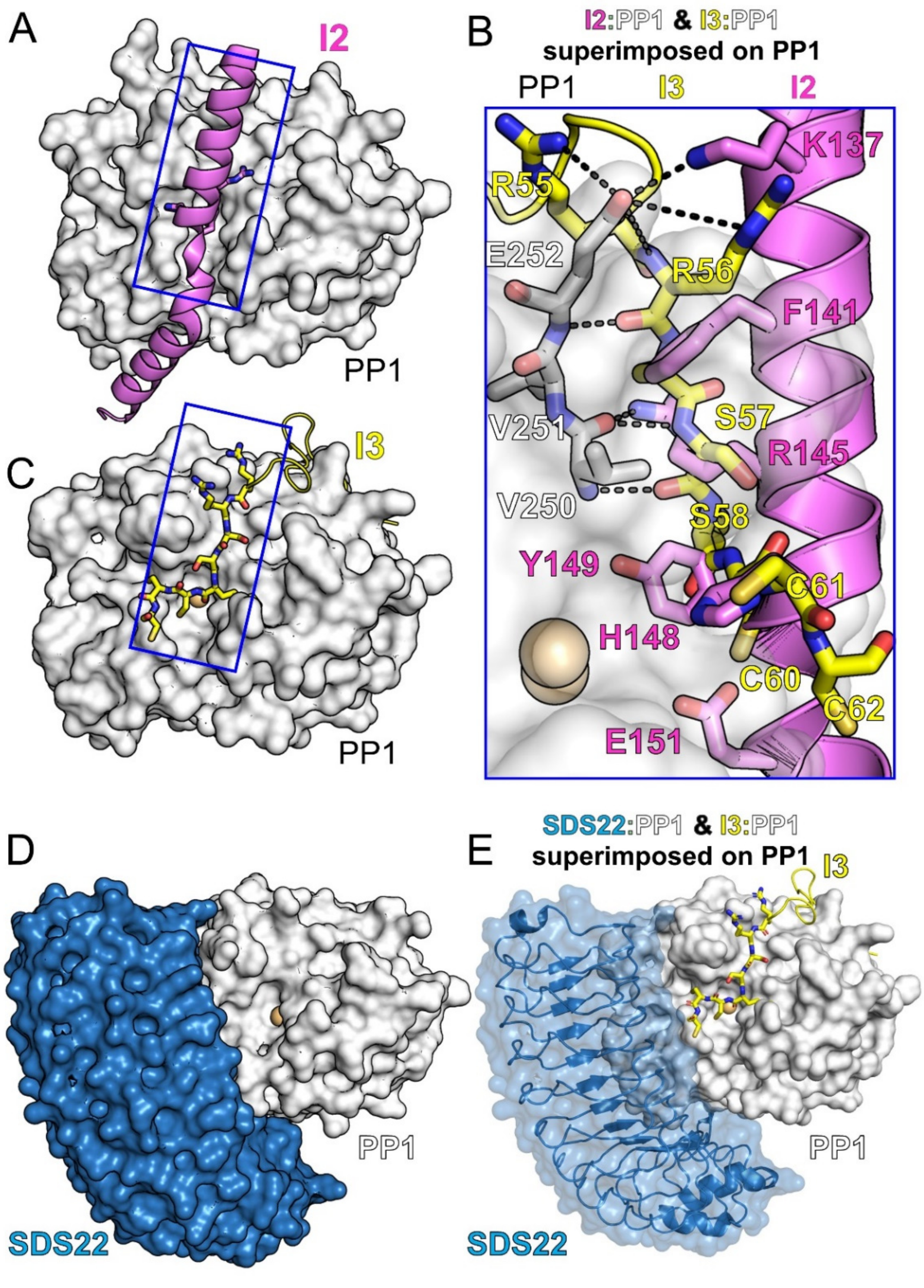

Supplementary Figure 6. Molecular mechanisms of PP1 inhibition. A. Structure of the I2:PP1 complex (PDBID 2O8G). PP1 is in grey and I2 in magenta; region highlighted by the blue box shown in (B). **B**. I3:PP1 (yellow:grey) and I2:PP1 (magenta:grey) complexes superimposed on PP1, illustrating the interactions of I3 and I2 in the PP1 acidic groove. Polar/electrostatic interactions are denoted by dashed, black lines. PP1 metals from the I3:PP1 comples shown as orange spheres. **C**. Structure of the I3:PP1 complex; PP1 is in grey and I3 in yellow; region highlighted by the blue box shown in (B). **D**. Structure of the SDS22:PP1 complex (PDBID 6OBN). PP1 is shown in grey and SDS22 in blue. **E**. The SDS22:PP1 and I3:PP1 complexes superimposed on PP1 illustrating that I3 and SDS22 do not clash.

Supplementary Table 1: SPR binding kinetics for the association of I3 variants with PP1α.

PP1 and I3 variants	$k_{on}1$ (10^5 M^{-1} s^{-1})	$k_{off}1$ (10^{-3} s^{-1})	$k_{on}2$ (10^{-3} s^{-1})	$k_{off}2$ (10^{-3} s^{-1})	K_D (nM)	χ^2	n
$PP1_{7-330}$ vs I3 variants							
**$I3_{1-126}$ (FL, full-length)**							
$I3_{1-126}$	4.1 ± 1.1	26.5 ± 4.7	8.5 ± 1.7	2.0 ± 0.4	15.7 ± 2	0.12 ± 0.05	3
$SILK_{dead}$: $I3_{1-126}$ T29A/I30A/K31A	1.3 ± 0.1	7.1 ± 2.1	9.4 ± 1.1	2.2 ± 0.1	13.1 ± 2.8	0.14 ± 0.02	3
$RVxF_{dead}$: $I3_{1-126}$ ^{40}KAEA^{43}	692.3 ± 61.3	23.8 ± 2.5	na	na	3460 ± 531	0.58 ± 0.27	4
SSS: $I3_{1-126}$ C60S/C61S/C62S	3.2 ± 0.1	97 ± 5.2	na	na	300 ± 23	-0.3 ± 0.1	3
**$I3_{27-68}$ (I3 PP1 interaction domain)**							
pH = 8.0	3.0 ± 0.3	21.8 ± 2.8	14.5 ± 1.8	1.9 ± 0.3	9.3 ± 1.1	0.12 ± 0.08	4
pH = 7.5	3.5 ± 0.2	24 ± 3	6.5 ± 1	0.9 ± 0.01	9 ± 0.6	0.19 ± 0.08	3
pH = 7.0	2.6 ± 0.3	28 ± 2.3	7.8 ± 1.2	1.1 ± 0.1	16.1 ± 1.1	0.16 ± 0.02	3
pH = 6.5	2.3 ± 0.1	27 ± 0.6	12 ± 1.4	1.8 ± 0.2	17 ± 0.8	0.33 ± 0.03	3
$SILK_{dead}$: $I3_{27-68}$ T29A/I30A/K31A	5.5 ± 0.2	42 ± 2.4	6.0 ± 0.4	1.3 ± 0.2	16.8 ± 2.8	0.14 ± 0.02	3
SCC: $I3_{27-68}$ C60S	1.8 ± 0.2	20.3 ± 2.4	13.3 ± 1.5	2.5 ± 0.2	21.7 ± 3.1	0.17 ± 0.03	5
CSC: $I3_{27-68}$ C61S	1.9 ± 0.1	32.1 ± 3.4	18.5 ± 1.7	2.1 ± 0.1	18.8 ± 0.9	0.14 ± 0.02	3
CCS: $I3_{27-68}$ C62S	2.5 ± 0.3	80.4 ± 20	18.3 ± 0.7	1.2 ± 0.1	20.6 ± 5.1	0.13 ± 0.04	3
CSS: $I3_{27-68}$ C61S/C62S	8.4 ± 0.5	16.8 ± 3.5	5.3 ± 1.4	5.5 ± 0.2	21.2 ± 0.6	0.28 ± 0.01	2
SCS: $I3_{27-68}$ C60S/C62S	6.2 ± 0.5	27.9 ± 1.5	4.3 ± 0.8	5.1 ± 0.4	54 ± 9	0.23 ± 0.04	3
SSC: $I3_{27-68}$ C60S/C61S	5.9 ± 0.3	51.4 ± 4.7	na	na	86.8 ± 3.8	0.26 ± 0.18	4
SSS: $I3_{27-68}$ C60S/C61S/C62S	1.8 ± 0.03	74 ± 2.2	na	na	412 ± 6	0.37 ± 0.11	3
IY: $I3_{27-68}$ I63A/Y64A	1.1 ± 0.01	19.4 ± 4.7	12.8 ± 1.2	1.2 ± 0.2	15.6 ± 2.4	0.18 ± 0.05	3

**$I3_{27-68}$ deletion variants**							
$SILK_{deletion}$: $I3_{38-68}$ (Δ27-37)	2.1 ± 0.2	17.3 ± 1.0	6.1 ± 1.0	1.1 ± 0.2	15.2 ± 2.5	0.39 ± 0.1	3
$CCC_{deletion}$: $I3_{27-59}$ (Δ60-68)	2.1 ± 0.3	76.6 ± 2.1	na	na	376 ± 47	0.49 ± 0.1	3
**$I3_{79-91}$**							
$I3_{79-91}$					No binding		3
**$I3_{27-68}$ vs. different PP1 variants**							
$PP1_{7-330}$ C273S	3.4 ± 0.2	20.9 ± 4	4.9 ± 0.6	1.1 ± 0.04	16.1 ± 0.7	0.22 ± 0.09	3
$PP1_{7-330}$ H66K	3.2 ± 0.2	12.8 ±1.8	11.9 ± 1.4	1.8 ± 0.2	5.9 ± 0.6	0.10 ± 0.01	3
$PP1_{7-330}$ H248N	2.1 ± 0.1	9.7 ± 0.4	11.7 ± 0.1	1.6 ± 0.4	6.6 ± 1.7	0.08 ± 0.01	2

na not applicable; bold indicates different I3 constructs or PP1 variants.

Supplementary Table 2. Data collection and refinement statistics

	$I3_{27\text{-}68}$:$PP1\alpha_{7\text{-}300}$ co-expressed[a]	$I3_{27\text{-}68}$:$PP1\alpha_{7\text{-}300}$ reconstituted[a]
PDBID	8DWL	8DWK
Beamline	SSRL 12-2	SSRL 12-2
Wavelength (Å)	0.97946	0.97946
Data collection		
Space group	$P4_12_12$	$P4_12_12$
Cell dimensions		
a, *b*, *c* (Å)	90.3, 90.3, 196.3	91.4, 91.4, 196.4
α, β, γ (°)	90.00, 90.00, 90.00	90.00, 90.00, 90.00
Resolution (Å)	2.00	2.50
R_{merge}	0.13 (3.07)	0.23 (2.31)
$I / \sigma I$	8.4 (0.5)	10.6 (1.5)
CC 1/2	0.99 (0.34)	0.99 (0.65)
Completeness (%)	99.7 (96.2)	99.9 (99.4)
Redundancy	8.0 (4.2)	12.1 (7.2)
Refinement		
Resolution (Å)	38.92-2.00 (2.02-2.00)	39.09-2.50 (2.54-2.50)
No. reflections	102249	54742
R_{work} / R_{free}	0.21 (0.44)/0.24 (0.40)	0.21 (0.32)/0.24 (0.32)
No. atoms		
Protein	5175	5124
Ligand/ion	12	4
Water	145	55
B-factors		
Protein	53.72	58.46
Ligand/ion	68.74	42.08
Water	49.00	50.49
R.m.s. deviations		
Bond lengths (Å)	0.04	0.002
Bond angles (°)	0.702	0.554
Ramachandran		
Outliers (%)	0.00	0.32
Allowed (%)	5.23	4.92
Favored (%)	94.77	94.76
Rotamer Outliers	3.67	3.21
Clashscore	3.40	3.06

[a]Data was collected from a single crystal
*Values in parentheses are for highest-resolution shell.

Supplementary Table 3: Apparent thermodynamic and dissociation constants for the Zn^{2+} interaction with $I3_{1\text{-}78}$ and $I3_{1\text{-}126}$ derived from ITC experiments at 25°C.

Interaction	K_D (μM)	ΔH (kcal·mol^{-1})	ΔS (cal.mol^{-1}.K^{-1})	-TΔS (kcal·mol^{-1})	ΔG (kcal·mol^{-1})	n
Zn^{2+}: $I3_{1\text{-}78}$	3.0 ± 0.4	-15.5 ± 2.0	-26.7 ± 6.8	7.9 ± 2.0	-7.5 ± 0.2	4
Zn^{2+}: $I3_{1\text{-}126}$	3.2 ± 0.7	-24.9 ± 6.4	-58.3 ± 21.8	17.4 ± 6.5	-7.5 ± 0.1	4
